# Heparanase inhibitor improves clinical study in patients with septic cardiomyopathy

**DOI:** 10.3389/fmed.2024.1429109

**Published:** 2024-08-07

**Authors:** Di Chen, Honglei Li, Shitao Huang, Zhongya Huang, Yibo Sun, Liping Liu

**Affiliations:** ^1^The First Clinical College of Lanzhou University, Lanzhou, Gansu, China; ^2^Department of Emergency Critical Care Medicine, The First Hospital of Lanzhou University, Lanzhou, China

**Keywords:** heart function, microcirculation, septic cardiomyopathy, heparanase inhibitor, mitochondrial function

## Abstract

**Objective:**

Septic cardiomyopathy (SCM), a prevalent and critical condition in individuals suffering from sepsis and septic shock, remains elusive in terms of its intricate pathogenesis, thereby lacking definitive diagnostic standards. Current clinical management predominantly revolves around addressing the underlying disease and alleviating symptoms, yet mortality rates persist at elevated levels. This research endeavors to delve into the effects of low molecular weight heparin on Heparanase (HPA) levels in SCM patients, while assessing the clinical significance of HPA as a diagnostic marker in this patient population.

**Method:**

A comprehensive cohort of 105 patients diagnosed with SCM was recruited from the Department of Critical Care Medicine at the First Hospital of Lanzhou University, spanning the period from September 2022 to October 2023, serving as the primary research subjects for this investigation. A prospective, randomized controlled trial was undertaken, wherein 53 SCM patients were randomly allocated to a control group receiving standard therapy, while 52 patients were randomly assigned to an intervention group receiving conventional treatment augmented with low molecular weight heparin (LMWH). On the 1st, 3rd, and 7th days post-treatment, the following parameters were measured and documented: HPA levels, syndecan-1 levels, IL-6, TNF-α, CD4+/CD8+ cell ratio, anti-Xa factor, antithrombin III (AT-III) levels, left ventricular ejection fraction (LVEF), fractional shortening (FS), E/e’ ratio, stroke volume (SV), cardiac performance index (CPI), global end-diastolic volume index (GEDVI), N-terminal pro-brain natriuretic peptide (NT-proBNP), cardiac troponin I (CTnI), heart-type fatty acid-binding protein (H-FABP), lactate (Lac) levels, central venous oxygen saturation (ScvO2), Sequential Organ Failure Assessment (SOFA) score, Acute Physiology and Chronic Health Evaluation II (APACHE II) score, ICU length of stay, and 28-day mortality rate.

**Results:**

In comparison to the control group, the LMWH group demonstrated significantly lower levels of HPA and syndecan-1 (*p* < 0.05), along with reduced levels of IL-6, TNF-α, E/e’, NT-proBNP, CTnI, H-FABP, GEDVI, SOFA score, APACHE II score, ICU length of stay, and 28-day mortality (*p* < 0.05). Additionally, the LMWH group exhibited increased levels of anti-Xa factor, AT-III, CD4+/CD8+ cell, LVEF, FS, SV, and CPI (*p* < 0.05). ROC curve analysis indicated that HPA can be combined with NT-proBNP, CTnI and H-FABP to improve the diagnostic efficiency of SCM.

**Conclusion:**

In SCM patient management, the integration of LMWH into conventional treatment significantly reduced HPA levels, mitigated syndecan-1 loss, attenuated inflammatory responses, enhanced immune function, improved microcirculation, cardiac systolic and diastolic functions, myocardial contractility, heart index, and end-diastolic volume. These interventions correlated with decreased clinical severity, ICU stays, and 28-day mortality rates in SCM patients.

**Clinical trial registration:**

https://www.chictr.org.cn.

## Introduction

Septic cardiomyopathy (SCM), a reversible cardiac dysfunction triggered by sepsis or septic shock, exhibits a wide incidence rate spanning from 10 to 70%, coupled with a notably high mortality rate (1). The contemporary clinical manifestations of septic cardiomyopathy (SCM) encompass: (1) left ventricular dilation coupled with normal or reduced filling pressure; (2) diminished ventricular contractility; and (3) right ventricular diastolic dysfunction or left ventricular systolic and/or diastolic dysfunction, concurrent with decreased volume responsiveness (2). The intricate pathogenesis of SCM primarily encompasses endocardial inflammatory damage, mitochondrial dysfunction, and microcirculation disorders, among other factors (1). Currently, the diagnosis of septic cardiomyopathy (SCM) predominantly relies on echocardiography-related parameters (e.g., LVEF, FS, E/e’) and hemodynamic monitoring indicators (SV, CPI, GEDVI). However, these methods are susceptible to human intervention and instrument detection errors, thereby limiting their use as the primary diagnostic reference for SCM (3). Furthermore, myocardial injury markers such as NT-proBNP, CTnI, and H-FABP serve as crucial biomarkers for SCM diagnosis. However, their sensitivity and specificity remain suboptimal and require validation through extensive clinical trials (1). Hence, the diagnostic criteria for SCM patients necessitate further investigation. Current SCM management primarily revolves around addressing the underlying disease, adequate fluid resuscitation, and the administration of vasoactive drugs (4). Consequently, additional research is imperative to elucidate SCM’s pathogenesis, standardize diagnostic criteria, and identify novel biomarkers, ultimately aiming to standardize treatment protocols and enhance patient outcomes.

Heparanase (HPA) is a heparin sulfate (HS)-specific glucuronidase that is a key structural component of the extracellular matrix (ECM) and is found in endothelial cells, monocytes and macrophages, and platelets (5). Inflammatory cytokines can activate HPA, which in turn catalyzes the shedding of endothelial glycocalyx (eGC) in cardiomyocytes, degrades HS fragments, increases the production of reactive oxygen species (ROS), and further leads to a vicious cycle of inflammation, causing myocardial dysfunction (6). HPA can directly activate the coagulation system, induce TF expression, increase factor Xa production, enhance coagulation activity, and lead to myocardial microcirculation disorder (7). Inhibiting HPA activity can protect the endothelial glycocalyx of cardiomyocytes, reduce inflammatory responses, improve microcirculation disorders, and further reduce the incidence of SCM (6). Therefore, the use of HPA inhibitors to reduce the inflammatory response and inhibit coagulation may become a clinical treatment method for patients with SCM.

Low molecular weight heparin (LMWH), a fragment derived from the cleavage of heparin, is extensively utilized as an anticoagulant owing to its antithrombotic properties (8). Its anticoagulant mechanism is primarily attributed to its capacity to bind with antithrombin III, thereby augmenting the inhibitory effect of AT-III on coagulation factor Xa (9). Furthermore, numerous studies have underscored LMWH’s anti-inflammatory properties. Hochart et al. previously demonstrated that LMWH reduces levels of proinflammatory cytokines and NF-κB in human monocytes stimulated by LPS, thereby inhibiting the inflammatory response (10). In 2021, Wu et al. conducted animal experiments demonstrating that LMWH ameliorates the inflammatory state in rats by inhibiting the TLR4-MyD88-NF-κB signaling pathway, suggesting a protective effect of LMWH against inflammatory diseases. However, the precise underlying mechanism remains to be elucidated (11). Sepsis is intricately linked to microthrombus formation and the activation of the coagulation system, often culminating in multiple organ dysfunction, particularly in severe cases. A recent meta-analysis revealed that LMWH effectively ameliorates multiple organ dysfunction syndrome in septic patients, subsequently reducing 28-day mortality rates (12). Meyer et al. hypothesized that heparin might enhance cardiac circulation and oxygen delivery in animals. Their study demonstrated that following 8 h of heparin administration in septic sheep, the heart index increased while systemic vascular resistance decreased (13). Concentrations of creatine kinase-MB (CK-MB) and homocysteine (Hcy) were notably elevated in the LPS-induced group. Conversely, following the administration of 100 IU/kg of low molecular weight heparin (LMWH) in the treatment group, circulating biomarkers were reduced, resulting in the inhibition of oxidative stress and the alleviation of heart damage. This finding further corroborates the protective efficacy of LMWH in cardiac dysfunction stemming from sepsis (14).

## Materials and methods

### Research subjects

A comprehensive cohort comprising a total of 105 SCM patients admitted to the Department of Critical Care Medicine at the First Hospital of Lanzhou University between September 2022 and October 2023 was selected for this study. The research protocol was approved by the hospital’s Medical Ethics Committee, and all participants provided informed consent prior to their inclusion in the study.

Inclusion criteria: (1) Age ≥ 18 years old, no gender limit; (2) Informed consent form of patients and their families; (3) Meet the diagnostic criteria of sepsis 3.0; (4) Echocardiography diagnostic index: left ventricular Ejection fraction (LVEF) < 50%, and/or left ventricular short-axis shortening (FS) < 30%; (5) Myocardial injury marker: CTnI > 1.5ug/L and NT-proBNP > 450 ng/L.

Exclusion criteria: (1) Suspected stress cardiomyopathy; (2) Patients with congenital heart disease, valvular heart disease, and recent acute myocardial infarction; (3) Patients with malignant tumors; (4) Patients expected to die within 24 h Patients; (5) Patients with bleeding disorders.

### Grouping and treatment

Patients who met the inclusion criteria were randomly assigned to the control or intervention group through stratified randomization generated by SAS statistical software. Each random number letter was prepared in duplicate and sealed in a blinded manner (randomization method). For statistical analysis, two blind exposures were performed, with the first blinding to divide the patients into groups and the second blinding to determine the specific drugs in each group. However, if the patient’s condition relapses or hemodynamic instability affects the patient’s prognosis during the study, the study will be terminated and the blinding will be lifted urgently. Maintenance of blindness: (1) Abide by the principle of priority of subjects’ rights and safety, do not compromise subjects’ rights and safety on the grounds of blind state maintenance, and perform emergency blinding if necessary; (2) Blind and non-blind team members should be relatively independent in their work, limit unnecessary communication, and avoid leaking blind information; (3) In addition to the routine records of clinical trials, the blind state maintenance process should be recorded accordingly, the subjects should be fully informed, and attention should be paid to the education work and the compliance of subjects. The patients were randomly divided into two groups: the control group:

The study comprised 53 patients in the control group, who received conventional treatment, and 52 patients in the LMWH group, who received LMWH treatment in addition to conventional therapy. The control group underwent necessary intensive care measures, encompassing mechanical ventilation, anti-infective therapy, vasopressor administration, fluid resuscitation, nutritional support, analgesia, and sedation. Meanwhile, the LMWH group received subcutaneous injections of LMWH (4000Uqd) for seven consecutive days, in addition to the aforementioned conventional treatment. The control group was administered subcutaneous injections of an equivalent dose of normal saline for seven consecutive days.

### Research parameters and experimental methodology

Baseline characteristics were collected for all patients, including age, gender, mean arterial pressure, myocardial injury markers, SOFA score, APACHE II score, ICU length of stay, and 28-day mortality rate. Echocardiography-related parameters (LVEF, FS, E/e’), hemodynamic monitoring indices (SV, CPI, GEDVI), and myocardial injury markers (NT-proBNP, CTnI) were observed on the 1st, 3rd, and 7th days post-treatment. Additionally, ScvO2, IL-6, anti-Xa factor, AT-III, CD4+/CD8+ cell ratio, and Lac levels were collected. Serum TNF-α, HPA, syndecan-1 and H-FABP were measured by enzyme-linked immunosorbent assay Enzyme-linked immunosorbent assay (ELISA).

The assay was performed using a kit provided by American Market Company. The day before the examination, take 4 mL of the patient’s cubital venous blood, add 1% of the total volume of EDTA, let it stand at room temperature for 20 min, centrifuge in a 4°C centrifuge for 15 min (1,000 g, 3,000 r/min), wash away the plasma, and distribute it in EP tubes, and store in a −80°C refrigerator for later use. Add 6 standard samples with concentrations of 0, 100, 250, 500, 1,000, and 2,500 pg./mL into the blank wells of the enzyme plate in sequence, with a volume of 100ul. Add 50ul of enzyme labeling solution to each well (excluding blank control wells); seal the ELISA plate with sealant and incubate it in a constant temperature water bath at 37°C for 1 h; rinse the ELISA plate and pat it evenly with absorbent paper; Add 50ul of revealing agent A and B 50 ul to each well; wrap it in tin foil and react in a dark place at 20–25°C for 10 min; add 50 mL of stop solution to the eac.

### Statistical analysis

Normally distributed data were expressed as mean ± standard deviation (SD) and compared with *t*-test. Non-normally distributed data were expressed as median (interquartile range, IQR) and compared using the Mann–Whitney U test. Count data were analyzed using χ^2^ test. The Kolmogorov–Smirnov test was used to test the normal distribution of the data. Analysis of variance (ANOVA) was performed on repeated measures of the general linear model. Correlations were analyzed using Pearson method. The Kaplan–Meier method was used to draw survival curves within 28 days after inclusion. The diagnostic value of HPA was evaluated by receiver operating characteristic (ROC) curve analysis. GraphPad Prism 8.0.2 software (SYSTAT, United States) was used to draw graphs. Statistical significance was determined using a *p*-value threshold of less than 0.05.

## Results

### Patient baseline characteristics

A random number method was used to divide 105 patients into the control group (*n* = 53) and the LMWH group (*n* = 52). The patient’s demographic data and clinical parameters on admission are shown in Table 1. The control group consisted of approximately 79% men and 20% women. 89% of patients in the LMWH group were male and 9% were female. There were no significant differences in body mass index, mean arterial pressure, serum HPA, serum CTnI and APACHE II scores between the two groups, but NT-proBNP was statistically significant between the two groups (*p* < 0.05).

**Table 1:** Baseline characteristics of patients in the control group and the LMWH group.

Parameter	Control (*n*=53)	LMWH (*n*=52)	*p*-value
Gender (%)			0.112
Male	42 (79.25)	47 (90.38)	
Female	11 (20.75)	5 (9.62)	
Age (median [IQR])	63.0 [56.0, 69.0]	63.0 [55.5, 70.5]	0.875
BMI (Kg/㎡)(median [IQR])	23.0 [21.6, 26.3]	24.4 [23.0, 26.5]	0.096
MAP (mmHg) (median [IQR])	92.0 (17.9)	93.5 (14.6)	0.644
Serum HPA (ng/ml) (median [IQR])	10.6 [9.6, 11.5]	10.1 [9.1, 10.9]	0.147
Serum CTnI (U/L) (median [IQR])	5.3 [3.6, 6.4]	5.2 [3.1, 6.2]	0.369
Serum NT-proBNP (pg/ml) (median [IQR])	2910.0 [1523.0, 5170.0]	1437.5 [1105.2, 4239.8]	0.025
APACHE II score (mean (SD))	15.0 [13.0, 17.0]	15.0 [12.8, 17.0]	0.687

HPA, heparanase; APACHE II, Acute Physiology and Chronic Health Score, APACHE II score ranges from 0 to 71; BMI, body mass index; MAP, mean arterial pressure; Serum HPA, serum heparanase; Serum CTnI, serum cardiac troponin I; Serum NT-proBNP, Serum N-terminal pro-B-type natriuretic peptide. Compare the *p* value between the control group and the group given low molecular weight heparin calcium.

### LMWH exhibits efficacious suppression of HPA expression and reduction in syndecan-1 and HPA levels

**Figure 1:**
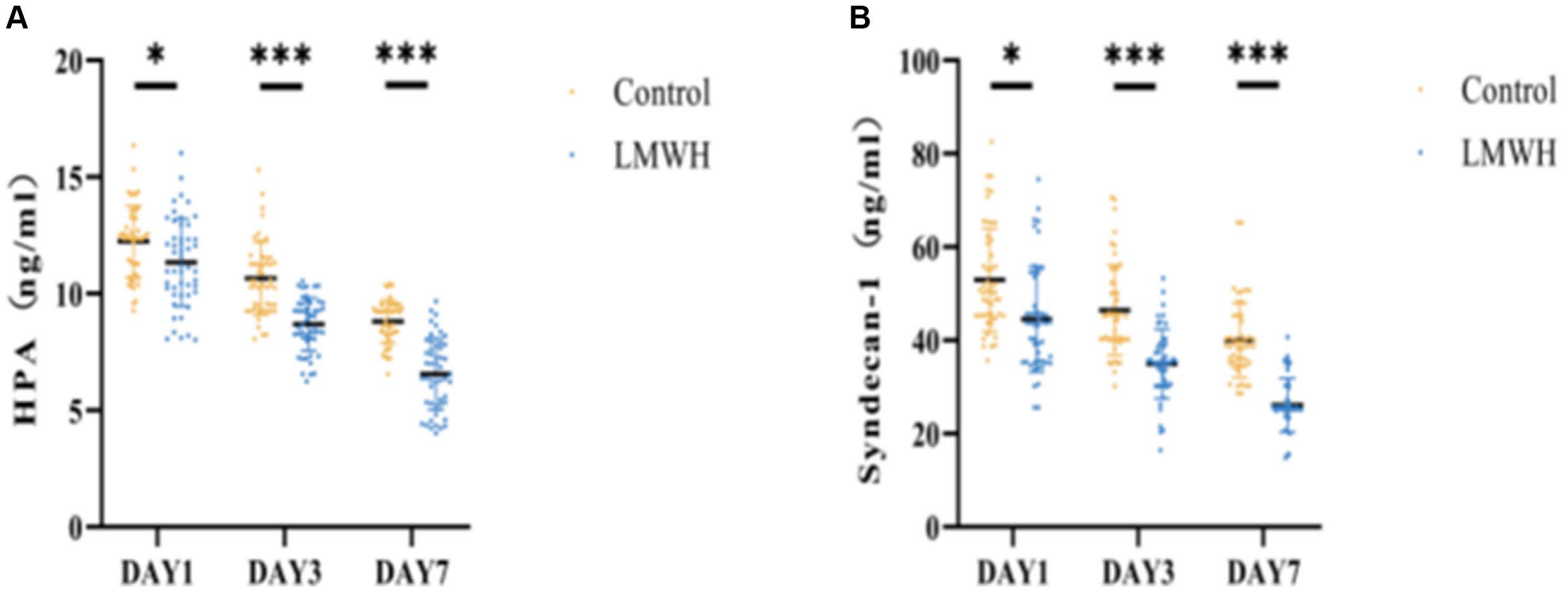
Comparison of two groups of HPA **(A)** and Syndecan-1 **(B)**. **(A)** HPA concentrations were compared between the two groups on days 1, 3, and 7. **(B)** Syndecan-1 concentrations were compared between the two groups on days 1, 3, and 7. HPA, heparinase; Syndecan-1, syndecan-1; *p < 0.05, ***p < 0.001.

Following treatment, the serum concentration of HPA was significantly elevated in the control group compared to the LMWH group (Figure 1A) (*p* < 0.05). Additionally, notable differences in serum syndecan-1 levels were observed between the two groups (Figure 1B) (*p* < 0.05). These findings suggest that the administration of LMWH effectively suppressed serum HPA and syndecan-1 levels in patients with SCM.

### The inhibitory effects of LMWH on inflammation, immunity, and coagulation functions in patients with SCM

**Figure 2:**
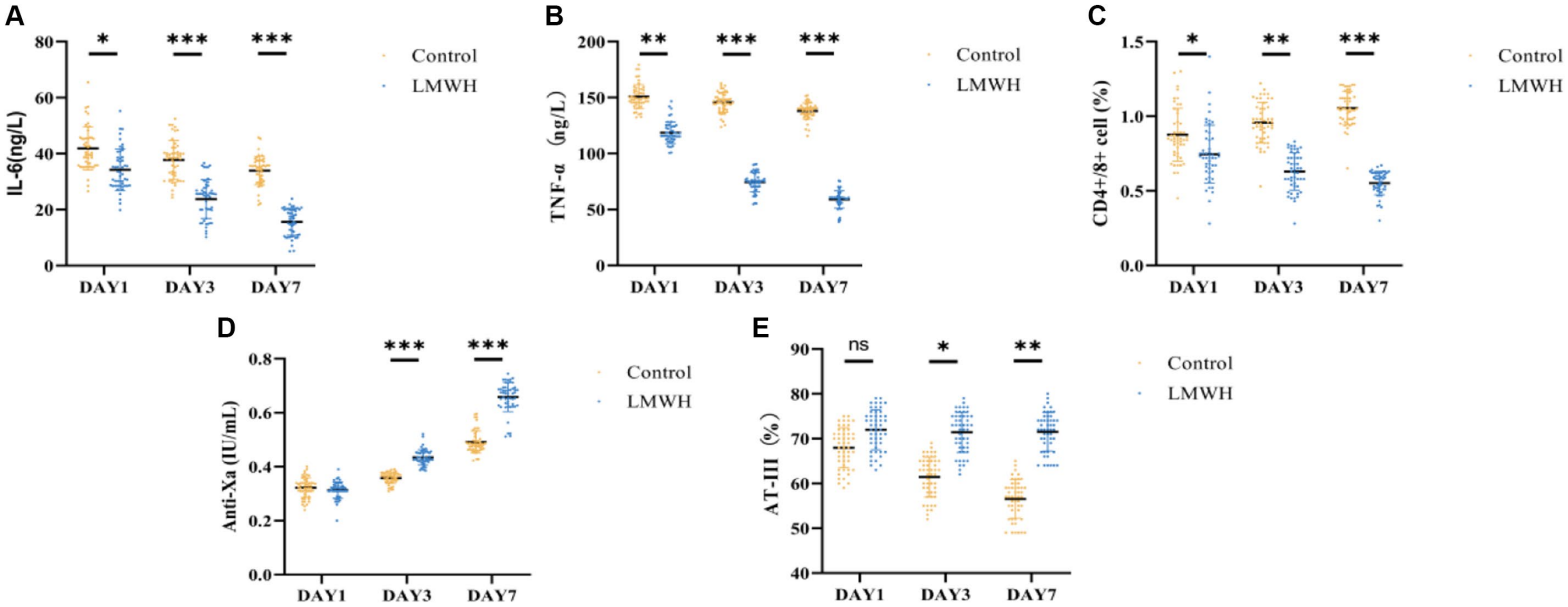
Comparison of IL-6 **(A)**, TNF-α **(B)**, CD4/CD8 **(C)**, anti-Xa factor **(D)**, and AT-III **(E)** between the two groups. **(A)** IL-6 concentrations were compared between the two groups on days 1, 3, and 7. **(B)** TNF-α concentrations were compared between the two groups on days 1 and 7. **(C)** Days 1, 3, and 7 compared the CD4+/CD8+ ratio between the two groups. **(D)** The anti-Xa factor concentration between the two groups was compared on the 1st, 3rd and 7th day; **(E)** The AT-III concentration between the two groups was compared on the 1st, 3rd, and 7th day. IL-6, interleukin 6; TNF-α, tumor necrosis factor-α; Anti-Xa, anti-Xa factor; AT-III, antithrombin-III. *p < 0.05, ***p < 0.001.

As depicted in Figures 2A,B, the serum levels of IL-6 and TNF-α in the LMWH group exhibited significant reductions on days 3 and 7 of treatment, compared to day 1 (*p* < 0.05). Following the 7th day of treatment, the anti-Xa factor level in the LMWH group increased significantly (*p* < 0.05) in comparison to the 1st day of treatment (Figure 2D), whereas the levels of AT-III were notably decreased (*p* < 0.05) (Figure 2E). Figure 2C illustrates that the LMWH group had a significantly higher CD4+/CD8+ cell ratio compared to the control group (*p* < 0.05). In summary, compared to the control group, the LMWH group exhibited superior anti-inflammatory, anticoagulant, and immune-enhancing effects.

### The administration of LMWH enhances cardiac systolic and diastolic functions, augments cardiac contractility, and increases cardiac index and global end-diastolic volume in patients with SCM

**Figure 3:**
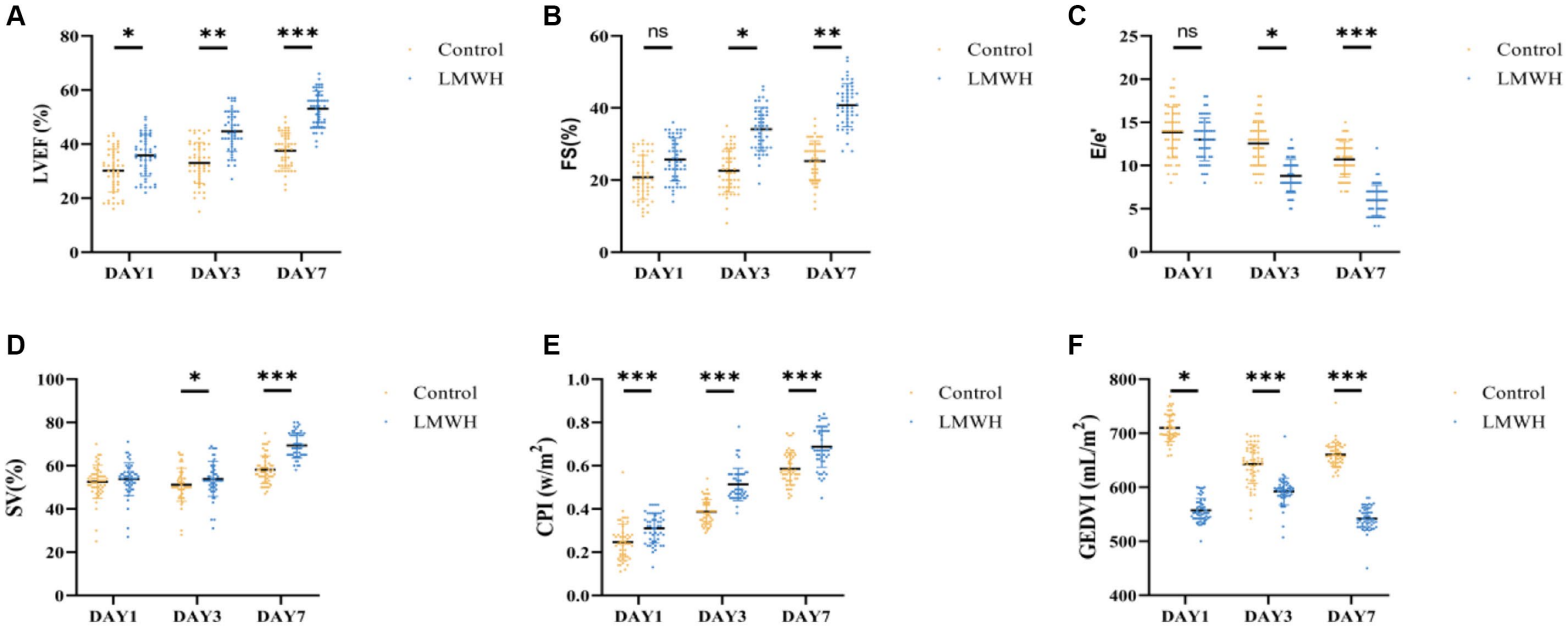
Comparison of LVEF **(A)**, FS **(B)**, E/e’ **(C)**, SV **(D)**, CPI **(E)**, and GEDVI **(F)** of the two groups. **(A)** LVEF values on days 1, 3, and 7 were compared between the two groups. **(B)** FS values were compared between the two groups on day 1, day 3, and day 7. **(C)** E/e’ values were compared between the two groups on days 1, 3, and 7. **(D)** SV changes between the two groups were compared on days 1, 3, and 7. **(E)** The CPI changes between the two groups were compared on days 1, 3, and 7. **(F)** The GEDVI changes between the two groups were compared on days 1, 3, and 7. LVEF, left ventricular ejection fraction; FS, short-axis shortening rate of the left ventricle; E/e’, early diastolic mitral valve blood flow/early diastolic mitral valve motion velocity value; SV, stroke volume; CPI, Cardiac index; GEDVI, global end-diastolic volume. *p < 0.05, ***p < 0.001.

As shown in Figures 3A,B, compared with day 1, the LVEF and FS levels of the LMWH group increased significantly on the 7th day of treatment (*p* < 0.05), and the E/e’ value of the LMWH group decreased significantly (*p* < 0.001) (Figure 3C). In terms of hemodynamic indicators, compared with day 1, the SV, CPI, and GEDVI values of the LMWH group increased on the 7th day of treatment (*p* < 0.001) (Figures 3D–F). It can be seen that compared with the control group, the LMWH group can improve the cardiac systolic and diastolic functions of SCM patients, and stabilize the cardiac contractility, cardiac index and global end-diastolic volume and other hemodynamic indicators of SCM patients. 4. The LMWH group can reduce the release of myocardial injury markers in SCM patients, and HPA can be used as a biomarker to protect SCM.

**Figure 4:**
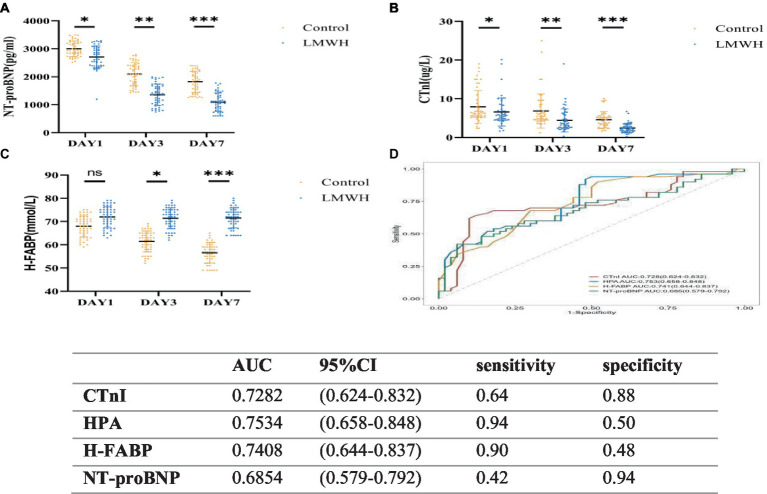
Receiver operating characteristic curves **(D)** of NT-proBNP **(A)**, CTnI **(B)**, H-FABP **(C)**, HPA, NT-proBNP, CTnI, and H-FABP in the two groups. **(A)** NT-proBN*p* values on days 1, 3, and 7 were compared between the two groups. **(B)** CTnI values were compared between the two groups on days 1, 3, and 7. **(C)** H-FABP values were compared between the two groups on days 1, 3, and 7. **(D)** Receiver operating characteristic curves of HPA, NT-proBNP, CTnI, and H-FABP. NT-proBNP, N-terminal precursor of brain natriuretic peptide; CTnI, cardiac troponin; H-FABP, heart-shaped fatty acid binding protein. *p < 0.05, ***p < 0.001.

As shown in Figures 4A–C, compared with day 1, the levels of NT-proBNP, CTnI, and H-FABP in the LMWH group were significantly reduced on days 3 and 7 of treatment (*p* < 0.05). As shown in Figure 4D, we plotted the ROC curves of HPA, NT-proBNP, CTnI, and H-FABP and calculated their AUC values. NT-proBNP, CTnI, and H-FABP are the traditional biomarkers of SCM. The AUC value of NT-proBNP is 0.685 with confidence interval of (0.579–0.792), and the AUC value of CTnI is 0.728 with confidence interval of (0.624–0.832). H-FABP has an AUC of 0.741 with a confidence interval of (0.644–0.837), but HPA has an AUC of 0.753 with a confidence interval of (0.658–0.848). In addition, the sensitivity of HPA is 0.94 and the specificity is 0.50, while the sensitivity of cTnI is 0.64 and the specificity is 0.88, the sensitivity of H-FABP is 0.90 and the specificity is 0.48, and the sensitivity of NT-proBNP is 0.42 and the specificity is 0.94. In conclusion, the sensitivity of HPA is the highest, while the specificity is not significant. HPA can be used as a biological factor to assist in the early diagnosis of SCM, but it still needs to be further confirmed in large-scale clinical studies.

### Administration of LMWH leads to improved Lac clearance and ScvO2 in patients with SCM

**Figure 5:**
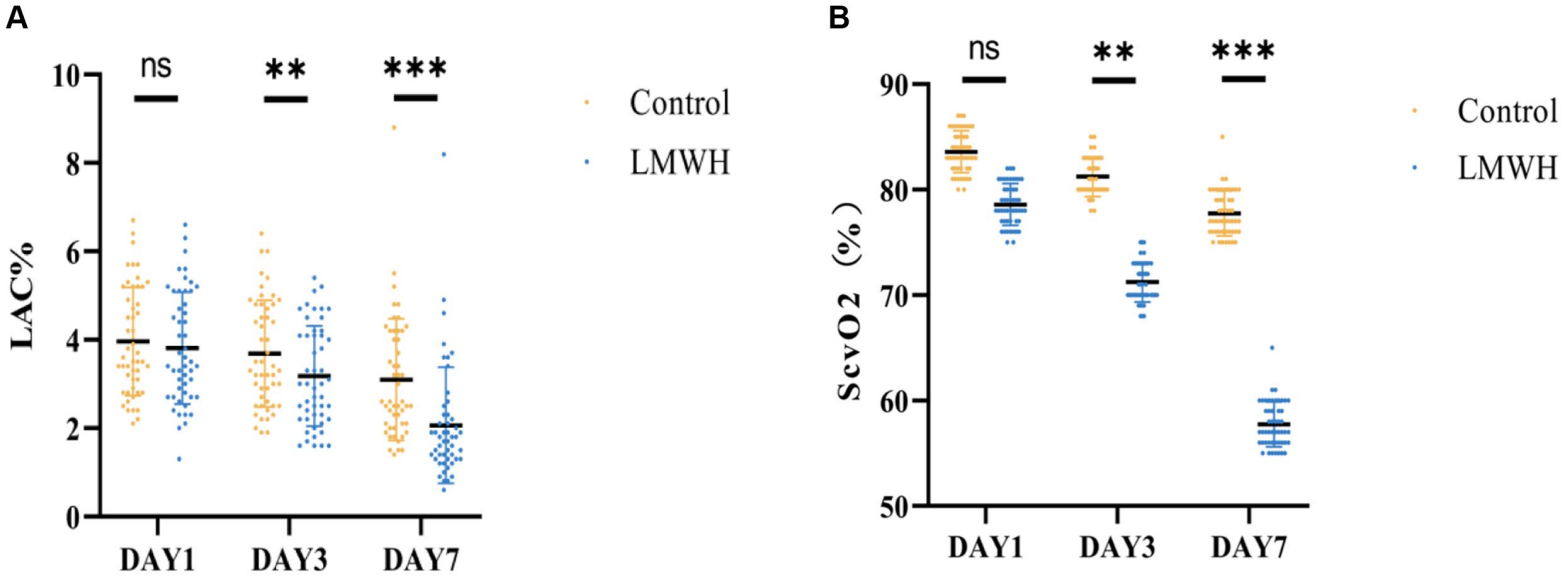
Two groups of Lac **(A)** and ScvO2 **(B)** are compared. **(A)** Lac values were compared between the two groups on days 1, 3, and 7. **(B)** ScvO2 values between the two groups were compared on days 1, 3, and 7. Lac, lactic acid; ScvO2, central venous oxygen saturation. *p < 0.05, ***p < 0.001.

As illustrated in Figures 5A,B, the lactate levels in the LMWH group were significantly lower on the 3rd and 7th days post-treatment compared to the control group (*p* < 0.05 and *p* < 0.001, respectively). In contrast, the ScvO2 levels in the LMWH group exhibited notable decreases on the 3rd and 7th days following treatment (*p* < 0.05 and *p* < 0.001, respectively).

### Administration of LMWH positively impacts the prognosis of septic cardiomyopathy patients, resulting in decreased ICU length of stay and 28-day mortality

**Figure 6:**
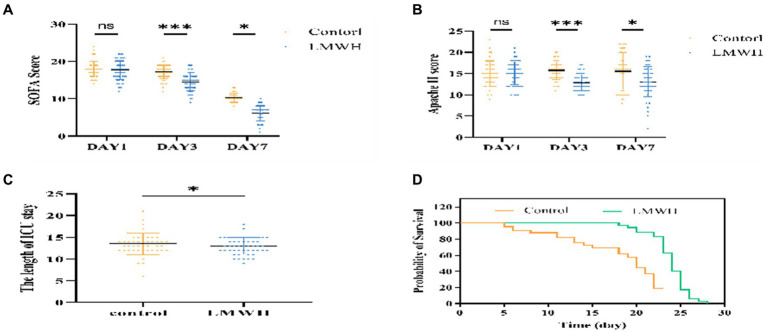
Comparison of SOFA score **(A)**, APACHE II score **(B)**, ICU length of stay **(C)**, Mortality rate within 28 days **(D)**. **(A)** Compare the changes in SOFA scores of the two groups of patients on days 1, 3, and 7. **(B)** Compare the changes in APACHE II scores between the two groups on days 1, 3, and 7. **(C)** Compare the ICU length of stay between the two groups. **(D)** Compare the 28-day mortality rate of the two groups. SOFA, Sequential Organ Failure Assessment; APACHE II, Acute Physiology and Chronic Health Evaluation II, The Length of ICU stay: ICU length of stay. *p < 0.05, ***p < 0.001.

As depicted in Figures 6A,B, on the initial day post-treatment, no significant differences were observed in the APACHE II and SOFA scores between the LMWH group and the control group. However, by the 3rd and 7th days post-treatment, both scores exhibited significant reductions in the LMWH group (*p* < 0.001 and *p* < 0.05, respectively). Figure 6C reveals a notably shorter duration of ICU stay in the LMWH group compared to the control group (*p* < 0.05). Furthermore, Figure 6D demonstrates a significantly lower 28-day mortality rate in the LMWH group compared to the control group (*p* < 0.001). In summary, LMWH demonstrates the potential to alleviate clinical severity, shorten ICU stay, and reduce 28-day mortality in patients suffering from septic cardiomyopathy.

## Discussion

In this experiment, we chose LMWH as a drug that inhibits HPA. The results show that inhibiting the expression of HPA can improve the cardiac systolic and diastolic functions of SCM patients and maintain the stability of hemodynamic indicators, reducing the release of myocardial injury markers and lactic acid and central venous oxygen saturation levels, which is beneficial to the patient’s prognosis. A meta-analysis showed that LMWH treatment in sepsis patients can reduce the expression of serum IL-6, TNF-α and other inflammatory factors, shorten prothrombin time, and improve coagulation (15). In terms of the prognosis of patients with sepsis, LMWH treatment significantly reduced the APACHE II score, thereby shortening the 28-day mortality of patients (16). In 1992, Frizelle et al. demonstrated that LMWH treatment can reduce collagen accumulation and inhibit myocardial inflammatory response in myocarditis animals, indicating that LMWH has anti-inflammatory effects (17). Microcirculation disorders are closely related to the pathogenesis of SCM. When ischemia and hypoxia occur, myocardial microvascular endothelium lacks anticoagulant properties, especially in the subendocardial layer. LMWH treatment can alleviate the ischemic response of microcirculation and prolong the survival time of patients (18). Therefore, we speculate that LMWH may improve the clinical symptoms and prognosis of SCM patients by protecting the endothelial glycocalyx, improving microcirculation, and inhibiting the expression of HPA (19).

A large number of studies have shown that HPA activity is significantly increased in sepsis-related organ damage, including acute respiratory distress syndrome (ARDS), acute kidney injury (AKI), and acute gastrointestinal dysfunction (AGI) (20–23). When sepsis occurs, the high expression of HPA leads to the release of a large number of inflammatory factors, enhances the interaction between immune cells and myocardial endothelial cells, and leads to circulating inflammatory responses (24), at the same time, HPA can damage the endothelial glycocalyx (eGC), increase vascular permeability, lead to interstitial edema, promote cytokine adhesion, and cause microcirculation disorders (25). Syndecan-1 is the main component of eGC. HPA can cleave the HS chain to degrade Syndecan-1. The shed HS chain can activate its own TLR-4, leading to a vicious cycle of inflammation (6). Therefore, we speculate that HPA and syndecan-1 may promote the occurrence and development of SCM. In our study, it was found that HPA and syndecan-1 were significantly elevated during the onset of SCM patients, and both were significantly reduced after treatment with LMWH. This finding is consistent with previous reports that LMWH improves outcomes in patients with acute gastrointestinal impairment in sepsis.

Studies have shown that traditional markers of myocardial injury, such as cTnI and NT-proBNP, are positively correlated with the severity of sepsis, and have nothing to do with myocardial damage (26). A multi-center retrospective clinical study of 184 patients with sepsis showed that cTnI was significantly higher in the death group than in the survival group, and similarly, cTnI concentrations were significantly higher in the SCM group than in the non-SCM group, demonstrating that cTnI can be an independent risk factor in SCM patients (27). In addition, when right heart dysfunction occurs in SCM patients, the plasma concentration of NT-proBNP is significantly increased, which may be due to the influence of pulmonary infection, acute respiratory distress syndrome, mechanical ventilation and other factors in the occurrence of sepsis, resulting in increased pulmonary vascular resistance, and fluid overload in SCM patients further aggravates the right ventricular load. The abnormal increase of NT-proBNP in SCM patients with right ventricular disorder increases the mortality of SCM patients (27). In 2012, Zhang et al. found that h-FABP is a biomarker that demonstrates mortality in patients with sepsis and septic shock and is associated with SCM (28). A recent prospective clinical study demonstrated that the concentration of h-FABP was significantly higher in the death group of patients with SCM than in the survival group, and the diagnostic ability of h-FABP was significantly higher than that of cTnI, but the accuracy needs further verification (29). In this study, CTnI, NT-proBNP and H-FABP were significantly reduced after LMWH treatment compared with the control group, indicating that the application of LMWH in SCM patients can reduce the release of myocardial injury markers. ROC curve analysis in this study showed that the sensitivity of HPA is the highest, while the specificity is not significant. HPA can be used as a biological factor to assist in the early diagnosis of SCM, but it still needs to be further confirmed in large-scale clinical studies. SCM patients exhibit myocardial inflammatory damage, immunosuppression, and microcirculatory dysfunction, and these symptoms are significantly improved after HPA inhibition. In addition, this study shows that LMWH combines with coagulation factor Xa to exert a stronger anticoagulant effect, AT-III is a physiological anticoagulant protein in the body, when combined with LMWH, it can inhibit the activity of various thrombin and coagulation factors, and is the main substance to prevent thrombosis (30). Therefore, in our study, the AT-III content in the LMWH group increased significantly with treatment time, and the anticoagulant effect was significantly better than that in the control group. The dose of LMWH used in this study has no associated risk of bleeding throughout the course of treatment, thus confirming that LMWH is a safe dose. In addition, we found that CD4+/CD8+ was significantly increased in the LMWH group (Figure 2C). It was once again confirmed that the inhibitory effect of LMWH on HPA can reduce myocardial inflammatory response, inhibit coagulation dysfunction and enhance immune function.

Echocardiography is considered an important modality in the overall assessment of patients with sepsis but remains challenging in diagnosing SCM. LVEF and FS are traditionally considered to be markers for diagnosing SCM. In this study, we found that the LVEF and FS values in SCM patients were significantly increased on the 7th day after LMWH treatment and the E/e’ value was decreased. Therefore, it can be confirmed that the application of LMWH to treat SCM patients can improve myocardial contractility and improve diastolic dysfunction (3). In addition, our study found that the SV and CPI of the LMWH group were significantly increased on the 7th day after treatment, and the GEDVI was significantly decreased on the 7th day after treatment, it was proved that LMWH treatment can improve left ventricular systolic force, strengthen cardiac blood supply, reduce cardiac load and alleviate the occurrence and development of SCM.

Lactic acid is the main oxidation source of the myocardium. Hyperlactatemia indicates tissue hypoperfusion (31). In this study, lactate levels in SCM patients decreased significantly after LMWH treatment, proving that LMWH can improve tissue perfusion in SCM patients. Some studies have shown that increased ScvO_2_ reflects insufficient oxygen utilization by tissues and may be related to mitochondrial dysfunction, further causing SCM and increasing mortality (32). After treatment with LMWH, the central venous oxygen saturation level of SCM patients significantly decreased, significantly improving patient prognosis. Furthermore, inhibition of HPA reduced clinical severity, ICU length of stay, and 28-day mortality in SCM patients. Although LMWH as an inhibitor of HPA has achieved clinical expected results in each group, there are still some errors that cannot be avoided, such as the influence of various confounding factors during the treatment process and changes in the patient’s condition, so more studies are still needed to prove it again as a result.

However, the shortcomings of this study are: (1) This study is a single-center study with a small number of patients. Multi-center studies still need to increase the sample size to further confirm the results; (2) The syndecan-1 measured in this study comes from plasma, but syndecan-1 exists in epithelial cells, but also originates from endothelial cells, which is difficult to determine the source; (3) In this study, we used LMWH a an HPA inhibitor, but no study has confirmed that it is a specific inhibitor of HPA. Therefore, there is still a need to seek HPA inhibitors with more specificity and higher clinical value.

## Conclusion

The results of this study show that LMWH can reduce the concentration of HPA, reduce the shedding of syndecan-1, reduce the inflammatory response, and protect the integrity of myocardial cell endothelium in patients with SCM. In addition, it can improve microcirculation disorders, enhance anticoagulant activity, improve myocardial systolic and diastolic function, stabilize hemodynamic indicators, reduce the release of myocardial injury markers, down-regulate Lac concentration and ScvO2 levels, and improve tissue hypoperfusion performance. In terms of prognosis, LMWH can delay the clinical severity, ICU length of stay, and 28-day mortality in SCM patients. In the future, HPA may become one of the biomarkers for early identification of SCM patients, and the combination with NT-proBNP, CTnI, and H-FABP can improve the diagnostic efficiency of SCM, and HPA inhibitors may also become a new target for the treatment of SCM.

## Data availability statement

The original contributions presented in the study are included in the article/supplementary material, further inquiries can be directed to the corresponding author.

## Ethics statement

The studies involving humans were approved by Ethics Committee of the First Hospital of Lanzhou University (Approval No. LDYYLL2024-49). The studies were conducted in accordance with the local legislation and institutional requirements. The participants provided their written informed consent to participate in this study.

## Author contributions

DC: Writing – original draft. HL: Writing – review & editing. SH: Investigation, Supervision, Writing – review & editing. ZH: Formal analysis, Methodology, Writing – review & editing. YS: Data curation, Writing – review & editing. LL: Investigation, Supervision, Writing – original draft.
